# A Victim of Dissipation

**Published:** 1875-12

**Authors:** 


### A VICTIM OF DISSIPATION.

Paul Morphy, the famous chess player, is in a New Orleans asylum hopelessly insane. He was born in that city in 1840, of wealthy Creole parentage, and his adoption of the game as a business, not only offended his relatives, but occupied the years in which he might have achieved success in some other career. He returned to his home suddenly and thoroughly disgusted with chess—so prejudiced against it that he has never since played. He has subsequently led an idle, morose life. His daily routine existence involved a walk through some of the streets of New Orleans every morning, where his dapper little figure—always scrupulously well dressed—became as well known and as regularly looked for as the noonday bell. After his daily promenade he retired from public gaze until evening, when he appeared in his box at the opera, where, it is said, he never missed a night. It is further related that during these years he permitted no friendly acquaintance; he was never known to associate with anybody but his mother, and persistently repelled advances from those who, having been friends of his early youth, desired to renew their associations. He lived a strange life, a strange, moody, and peculiarly mournful man. About a year ago he began to lose his mental control, and several months ago was put in a private asylum. Some of his friends hold the theory that his malady had its start in the strain upon his mind in playing many and difficult games of chess. This theory is not sufficient to account for his loss of reason. He was simply the victim of dissipation. Not, so far as we know, of the kind of dissipation which involves hard-drinking and late hours, but of the sort which gives out or dissipates without taking in anything. Steady, attention earnestly devoted to speaking or writing, or playing chess, without acquisition, without taking in something to make up for the outgo, always results in insanity or death.

---

Sure Death—Hot alum-water is a suggestion for destroying red and black ants, cockroaches, spiders, chintz-bugs, and all the crawling pests which infest our houses. Take two pounds of alum, and dissolve it in three or four quarts of boiling water; let it stand on the fire until the alum disappears; then apply it with a brush, while nearly boiling hot, to every joint and crevice in your closets, bedsteads, pantry-shelves, and the like. Brush the crevices in the floor of the skirting or mop-boards, if you suspect they harbor vermin. If, in whitewashing a ceiling, plenty of alum is added to the lime, it will also serve to keep insects at a distance. Cockroaches will flee the paint which has been washed in cool alum-water. Sugar-barrels and boxes can be freed from ants by drawing a wide chalk mark just around the edge of the top of them. The mark must be unbroken, or they will creep over it; but a continuous chalk-mark half an inch in width will set their depredations at naught. Powdered alum or borax will keep bedbugs at a respectful distance; and travellers should always carry a package of it, to scatter in the bed in places where they have reason to suspect the presence of such bedfellows.

